# Intracellular Proton Access in a Cl^−^/H^+^ Antiporter

**DOI:** 10.1371/journal.pbio.1001441

**Published:** 2012-12-11

**Authors:** Hyun-Ho Lim, Tania Shane, Christopher Miller

**Affiliations:** Department of Biochemistry, Howard Hughes Medical Institute, Brandeis University, Waltham, Massachusetts, United States of America; University of Zurich, Switzerland

## Abstract

Chloride-transporting membrane proteins of the CLC family appear in two distinct mechanistic flavors: H^+^-gated Cl^−^ channels and Cl^−^/H^+^ antiporters. Transmembrane H^+^ movement is an essential feature of both types of CLC. X-ray crystal structures of CLC antiporters show the Cl^−^ ion pathway through these proteins, but the H^+^ pathway is known only inferentially by two conserved glutamate residues that act as way-stations for H^+^ in its path through the protein. The extracellular-facing H^+^ transfer glutamate becomes directly exposed to aqueous solution during the transport cycle, but the intracellular glutamate E203, Glu_in_, is buried within the protein. Two regions, denoted “polar” and “interfacial,” at the intracellular surface of the bacterial antiporter CLC-ec1 are examined here as possible pathways by which intracellular aqueous protons gain access to Glu_in_. Mutations at multiple residues of the polar region have little effect on antiport rates. In contrast, mutation of E202, a conserved glutamate at the protein–water boundary of the interfacial region, leads to severe slowing of the Cl^−^/H^+^ antiport rate. An X-ray crystal structure of E202Y, the most strongly inhibited of these substitutions, shows an aqueous portal leading to Glu_in_ physically blocked by cross-subunit interactions; moreover, this mutation has only minimal effect on a monomeric CLC variant, which necessarily lacks such interactions. The several lines of experiments presented argue that E202 acts as a water-organizer that creates a proton conduit connecting intracellular solvent with Glu_in_.

## Abstract 2

Mutagenesis, functional analysis, and crystal structures identify a watery tunnel through which protons enter the interior of a Cl^−^/H^+^ antiport protein involved in acid resistance of enteric bacteria.

## Author Summary

Chloride-proton antiport proteins of the “CLC” superfamily are transmembrane proteins that form homodimers and are used for myriad physiological purposes, all requiring the coordinated movements of Cl^−^ anions and H^+^ cations in opposite directions across biological membranes. While the pathway for Cl^−^ ions through CLC antiporters is known, we currently have only indirect glimpses of how protons navigate their way through these membrane-embedded proteins. By combining mechanistic and structural approaches, we identify a proton-access pathway in a bacterial Cl^−^/H^+^ antiporter that allows intracellular protons to enter the protein interior and engage in the coupled antiport mechanism. We conclude that E202, a highly conserved glutamate residue, serves to organize water molecules and guide protons to the adjacent glutamate E203 (known as “Glu_in_”), a critical residue for the antiport mechanism.

## Introduction

**Figure 1:**
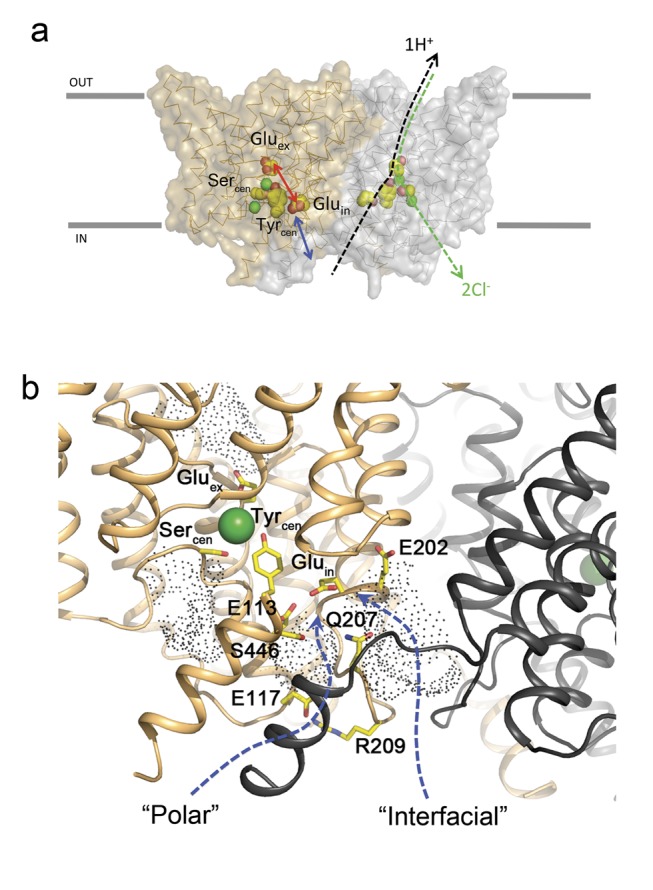
Ion transport pathways and solvent accessibility of CLC-ec1. (a) CLC-ec1 (PDB #1OTS) is shown in surface view, with subunits of the homodimer differently colored and drawn to indicate different aspects of the antiport mechanism. Key mechanistic residues are space-filled. Bifurcated Cl^−^ and H^+^ pathways are indicated as dashed lines on right subunit. Separation of Glu_in_ from the intracellular solution is shown with a blue arrow, central region between Glu_in_ and Glu_ex_ with a red arrow, and the internal and central Cl^−^ ions as green spheres. In subsequent figures, the internal Cl^−^ ion is omitted, since this binds weakly and is unlikely to be directly involved in the transport mechanism. (b) Close-up view of the intracellular surface of CLC-ec1 near Glu_in_. Aqueous clefts are shown as dots, and the twin subunit is shown in greyscale to visualize the subunit interface. Polar and interfacial pathways—possible routes for H^+^ access to Glu_in_—are indicated with arrows.

Proton-coupled anion exchange-transporters of the CLC family carry out varied physiological tasks in virtually all eukaryotes and many prokaryotes, transporting Cl^−^, NO_3_^−^, or F^−^ across membranes in strictly coupled exchange for H^+^ ions in the opposite direction [1],[2]. These transporters can thus use a proton gradient to pump anions thermodynamically uphill or vice versa, depending on biological context. CLC proteins are membrane-embedded homodimers in which each subunit acts independently as a functional unit [3]–[8]. The pathways within each subunit taken by Cl^−^ and H^+^ have been delineated mainly by studies of a homologue from *Escherichia coli*, CLC-ec1, which is uniquely tractable at levels of biochemistry [9], electrophysiology [10],[11], and X-ray crystallography [12]. The H^+^ and Cl^−^ pathways (Figure 1a) run together on the extracellular side of the protein, diverging about halfway through, where a “central” Cl^−^ ion resides in the ion-coupling chamber [13]. A critical “external glutamate,” E148 (Glu_ex_), serves two mechanistically essential purposes. It forms an extracellular gate that closes or opens the ion pathways to the extracellular solution via side-chain rotation between buried and water-exposed positions and, coupled to this structural change, transfers H^+^ between protein and solution [12]. If this residue is substituted with a nonprotonatable group, H^+^ transport is completely abolished while Cl^−^ movement, now uncoupled, persists [10]. A second key “internal glutamate” residue located towards the intracellular side of the protein, E203 (Glu_in_), similarly mediates H^+^ transfer between protein and the internal solution; mutation of this residue to nonprotonatable moieties also abolishes H^+^ movement while preserving Cl^−^ transport [13]. Unlike Glu_ex_, Glu_in_ need not physically move to hand off its proton [11], but merely places a dissociable group at this location in a proton-transfer pathway.

While the Cl^−^ pathway is crystallographically visible, the H^+^ pathway has been glimpsed only indirectly through the two carboxylate way-stations visited during the transport cycle, Glu_ex_ and Glu_in_. These groups are located ∼15 Å apart, separated by a nonpolar—and in the crystal structure anhydrous—region containing only a single dissociable side chain: a tyrosine residue whose hydroxyl group may be removed without disruption of transport [14], indicating its nonessential role. Moreover, while Glu_ex_ directly communicates with extracellular aqueous protons in its open position, Glu_in_ is buried away from the internal solution by a protein layer ∼10 Å thick (Figure 1a). Thus, two basic questions regarding the H^+^ pathway remain unresolved: (1) how do intracellular aqueous protons gain access to Glu_in_, and (2) how do protons negotiate the nonpolar desert interposed between Glu_in_ and Glu_ex_?

This study addresses the first of these questions by examining two potential access pathways through mutations that impair H^+^ transport. We find that intracellular H^+^ access to Glu_in_ can be greatly slowed—indeed, made rate-limiting for Cl^−^ antiport—by blocking one of these portals with mutation of E202, a strongly conserved residue of hitherto unknown function that guides aqueous H^+^ to several water molecules positioned in proximity to the carboxylate moiety of Glu_in_.

## Results

The absence of polar groups between Glu_in_ and Glu_ex_ suggests strongly that water wires catalyzing proton transfer somehow connect these essential carboxyl groups during the transport cycle. Previous computational studies [15]–[18] have arrived at pictures of such water-based H^+^ pathways, similar in theme but varied in detail. While it is widely thought from the physical character of CLC proteins that water-chains must be involved in H^+^ transfer between the two critical glutamates, such waters are neither experimentally manipulable by currently available tools nor crystallographically visible. However, the other unknown feature of the proton pathway—proton access to Glu_in_ from intracellular solution—is susceptible to experimental inquiry.

The unusually high Cl^−^ transport rate of CLC-ec1, 2000–3000 s^−1^ [14],[19], makes it natural to wonder, since Glu_in_ is buried, whether cytoplasmic H^+^ access to Glu_in_ is a rate-determining step in the transport cycle, and if not, why not. To obtain a sharper picture of this region in hopes of locating waters more prominent than in the original high-quality 2.5 Å crystal structure [12], we engineered CLC-ec1 guided by its crystal-packing interfaces, deleting 15 N-terminal and four C-terminal residues. This construct, denoted ΔNC, removes residues either disordered or forming problematic crystal contacts in previous structures and produced high-quality electron density maps from crystals routinely diffracting to 2.2–2.7 Å Bragg spacing (Table S1). This trimmed construct is essentially identical to wild-type protein in structure, absolute turnover rate, and exchange stoichiometry (Figure S1a,b, Table S2). Henceforth, we use a ΔNC dataset refined to 2.5 Å for structural examination and full-length proteins for functional analysis. We confidently localized many fewer water molecules than were modeled in the original structure (52 versus 167, exclusive of the Fab fragment), but the number and locations of waters near Glu_in_ in both structures match well (Figure S2a).

Inspection of the transporter's intracellular surface reveals two watery clefts that might potentially allow protons access to Glu_in_ (Figure 1b). One of these—the “polar pathway”—contains five crystallographically visible waters embedded within a cluster of polar side chains, some of which form salt bridges (Figure S2b,c). The other—the “interfacial pathway”—is a narrow, aqueous invagination, or fjord, between the two CLC subunits topped by a conserved glutamate, E202. As described in the Supporting Information section (Text S1, Figure S3, Table S2), our attempts to disrupt the polar pathway by removing salt bridges, eliminating charges, or replacing polar side chains with hydrophobics caused only unimpressive inhibition of H^+^ transport and impaired Cl^−^/H^+^ coupling minimally or not at all.

We therefore turned our attention to the interfacial pathway, focusing in particular upon E202, for several reasons. First, this residue forms part of the protein surface that separates bulk water in the interfacial fjord from the protein interior where Glu_in_ is buried. Second, a network of crystallographic water molecules near E202 and Glu_in_, including one bridging their carboxylates, invites closer examination of E202's role in the transport cycle (Figure S2d). Third, E202 is arguably the most strictly conserved residue in the CLC superfamily, and yet its function is entirely unknown.

**Figure 2:**
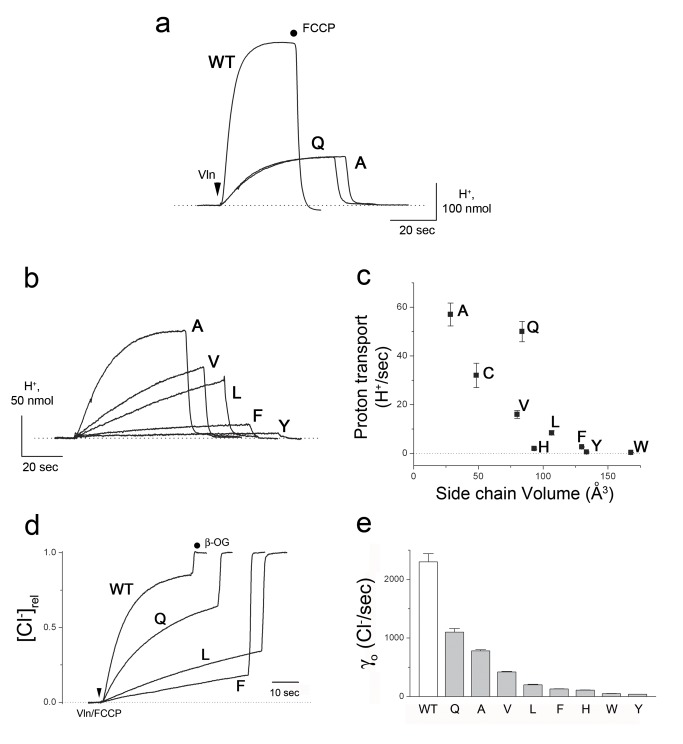
Effect of E202 mutations on Cl^−^-driven H^+^ uptake. (a) H^+^ uptake traces of E202A and E202Q. Transport is initiated by addition of Vln (arrow) and terminated by FCCP, and H^+^ uptake is indicated by upward deflection in the pH traces shown. (b) H^+^ uptake traces with indicated substitutions at E202. (c) Correlation of initial H^+^ uptake rate with side chain volume. (d) Cl^−^ efflux traces with indicated substitutions at E202. Cl^−^ appearance in the liposome suspension is normalized to final value after addition of β-octylglucoside detergent (after abrupt step in trace). Arrow indicates addition of Vln+FCCP. (e) Summary of initial rates of Cl^−^ efflux, γ_o_, for E202 substitutions tested.

Since our concern is with H^+^ access, and because we have no direct measurement of H^+^-binding kinetics to Glu_in_, we examined the rate of H^+^ pumping driven by a Cl^−^ gradient. CLC-ec1-reconstituted liposomes loaded with 300 mM KCl were diluted into a lightly buffered solution containing 10 mM Cl^−^ and 300 mM K^+^, and the pH of the suspension was monitored continuously. Addition of the K^+^ ionophore valinomycin (Vln) initiates transport by electrically shunting Cl^−^/H^+^ exchange and setting the liposome membrane potential to zero. Two nonprotonatable mutants, E202Q and E202A, the former isosteric and polar and the latter small and nonpolar, were first tested (Figure 2a) and found to support H^+^ uptake at rates about 20% of WT CLC-ec1. This result demonstrates that protonation of E202 is not required for antiport, an unsurprising conclusion since E202Q has been long known to maintain near-wild-type Cl^−^/H^+^ exchange stoichiometry [13]. Despite its strict conservation, E202 may be substituted with many other side chains without impairing protein expression, folding, or dimerization, and so we tested a series of nondissociable side chains at this position. A clear pattern emerges (Figure 2b,c) in which H^+^ transport slows as side-chain volume increases, with the large aromatics producing up to 200-fold inhibition of the H^+^ uptake rate. In these very slow mutants, the antiport mechanism remains intact, as shown by measurements of Cl^−^ efflux, which slows down in parallel, although not to the same extent as H^+^ uptake (Figure 2d,e). All rates are reported in Table S2.

**Figure 3:**
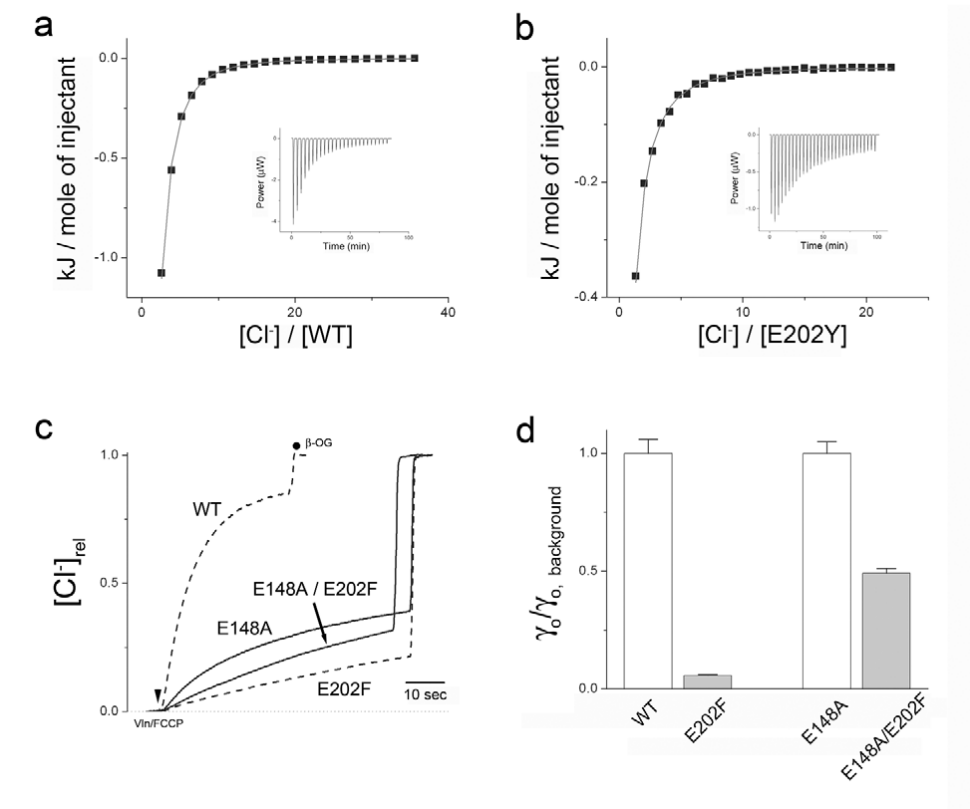
Effect of E202 substitutions on Cl^−^ pathway. (a, b) Equilibrium Cl^−^ binding isotherms determined by ITC for wild type or E202Y, respectively. Solid curves represent single-site binding curves with K_D_ = 0.74 and 1.6 mM, respectively (Table S3). (c) Effect of E202F mutation on Cl^−^ efflux traces in fully H^+^-uncoupled transporter, E148A. For comparison, dashed lines reprise the effect of E202F on the H^+^-coupled wild type. (d) Summary of E202F effect on Cl^−^ efflux rates γ_o_ on coupled (WT) and uncoupled (E148A) backgrounds. Rates are normalized to the background value for comparison.

It is tempting to imagine that the severe inhibition of H^+^ transport by these aromatic substitutions at E202 reflects blockage of the path from cytoplasmic water to Glu_in_, such that H^+^ access becomes rate-limiting for the transport cycle. But this conclusion would be invalid without evidence that these mutations act upon H^+^ movement itself, rather than on Cl^−^ transport, the observed inhibition of H^+^ uptake being merely a secondary consequence of the coupled antiport mechanism. The question thus becomes: Which ion's pathway is the primary victim of the mutations? We address this question by examining two diagnostics of the Cl^−^ pathway that are independent of H^+^ involvement: Cl^−^ binding and uncoupled Cl^−^ transport. Equilibrium binding of Cl^−^ indicates that the E202Y mutation does not act directly on the Cl^−^ transport pathway, since Cl^−^ affinity determined by isothermal calorimetry, known to reflect binding to the central anion site [19],[20], is weakened only 2-fold in E202Y (Figure 3a,b, Table S3). These mutations are further tested for Cl^−^ transport in a CLC mutant (E148A) wherein all H^+^ transport is eliminated [10]. This mutant, lacking the external Glu_ex_ gate and devoid of acid activation and H^+^ coupling, provides a way of testing the effect of E202 mutants on the Cl^−^ pathway alone. The question is straightforward: Do large nonpolar substitutions at E202 severely inhibit Cl^−^ transport on a background of E148A, as they do on wild-type? The answer is clear: they do not (Figure 3c,d, Figure S4). The Cl^−^ efflux rate is altered by trivial factors of 0.5, 1.7, and 1.9 for the F, Y, and W substitutions, respectively. A similarly minimal effect of the F substitution is also seen on a different H^+^-uncoupled mutant background (E148A/Y445S, Figure S4) with a 20-fold faster Cl^−^ turnover rate than wild-type [21]. These several lines of evidence argue that large, nonpolar side chains at E202 slow the coupled transport cycle not by impairing the Cl^−^ transport pathway but rather by rendering H^+^ diffusion between Glu_in_ and intracellular solution rate-limiting.

**Figure 4:**
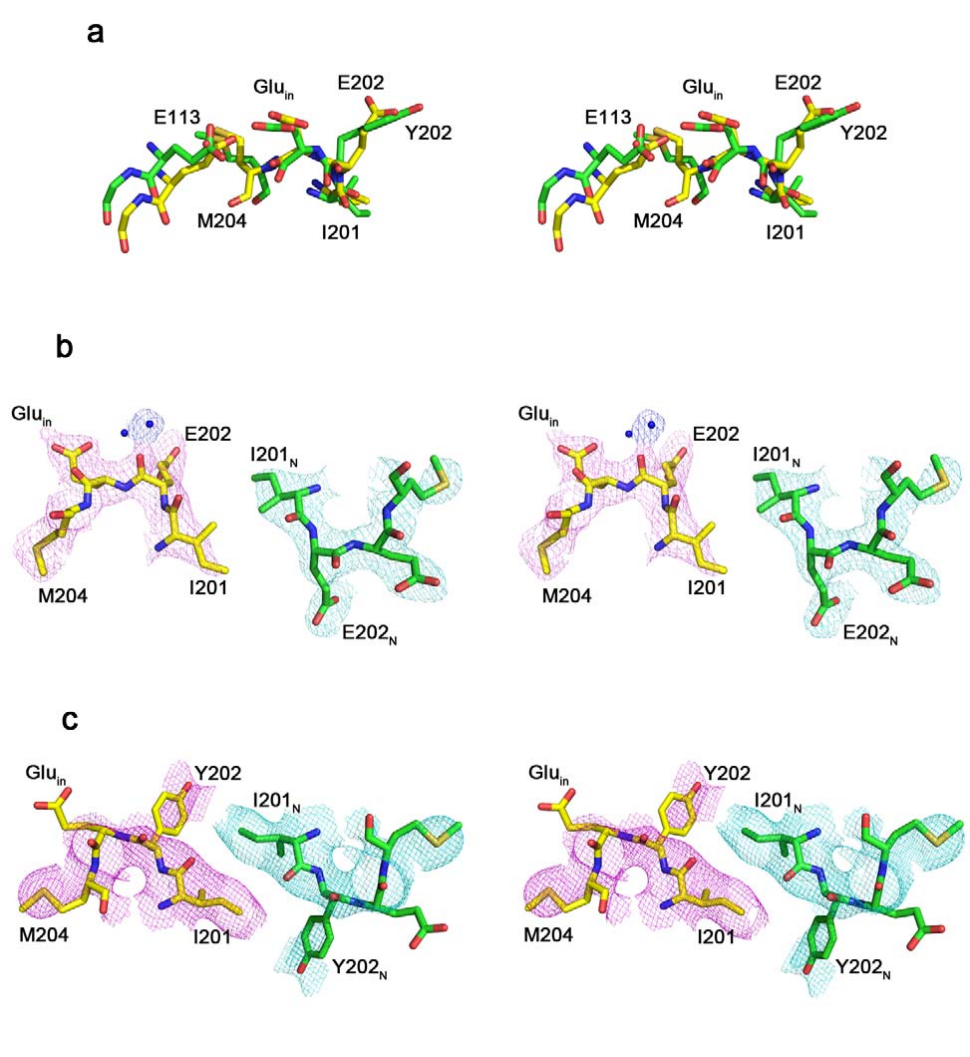
Structural changes in E202Y. The mutant backbone is basically unaltered from wild type (Cα rmsd 0.9 Å). (a) Structural comparison between wild-type (ΔNC) and E202Y mutant near the E202 residue. Residues are colored in *yellow* (for wild type) or *green* (for E202Y), *red* (oxygen), and *blue* (nitrogen). Cytoplasmic side view into the apex region of the interfacial pathway for wild type (b) and E202Y (c). E202_N_ and I201_N_ indicate residues coming from the neighboring subunit of the homodimer. Crystallographic water molecules are shown in *blue* dots. 2F_o_-F_c_ maps are contoured at 1.0 σ.

By what mechanism do these E202 substitutions so strongly inhibit H^+^ transport? Though unable to grow crystals of any E202 mutant suitable for assessing water organization near this residue, we obtained a single dataset of E202Y at a resolution sufficient (3.2 Å) to observe the disposition of this inhibitory side chain. The mutant is essentially identical in structure to wild-type protein and shows prominent density at the central Cl^−^ binding site (Figure S5). The substituted side chain adopts the position that would otherwise be a polar portal at the top of the fjord, with the phenol ring bricking up that gateway with greasy mortar (Figure 4a). Moreover, an unexpected consequence of this mutation is the movement of the I201 side chain from the neighboring subunit to pack closely against the Y202 aromatic ring, a subtle cross-subunit rearrangement that contributes additional nonpolar mass to sequester Glu_in_ away from aqueous protons in the interfacial fjord (Figure 4b,c). Thus, even at this rather low resolution, the E202Y structure neatly rationalizes the dramatic slowdown of H^+^ transport suffered by the large nonpolar E202 mutants.

**Figure 5:**
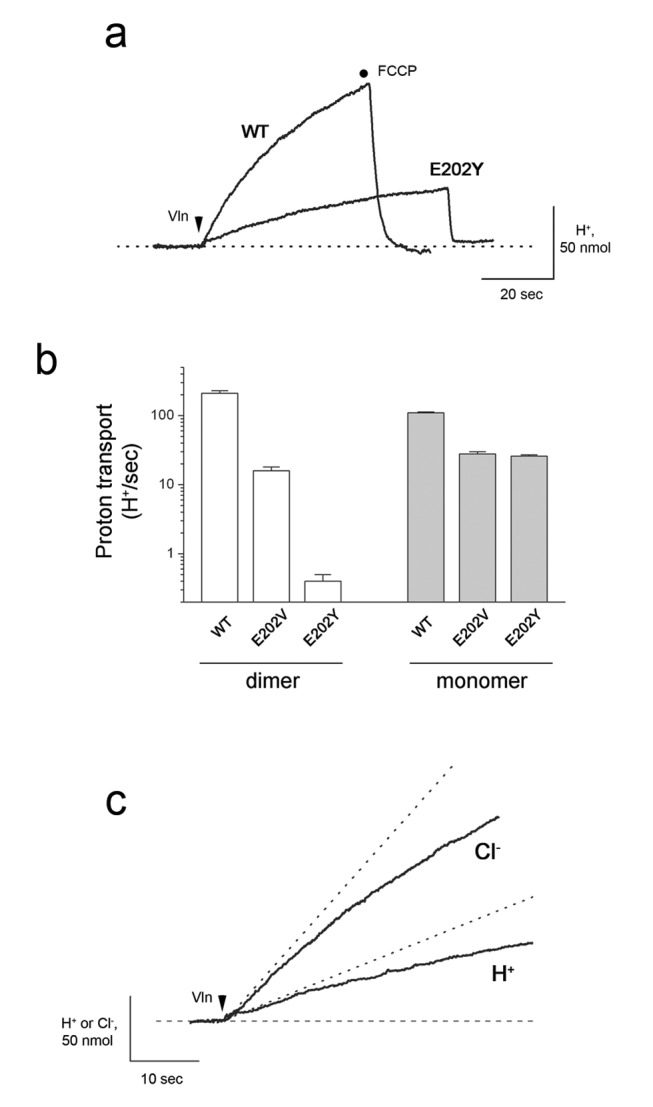
Test of E202 mechanism in a monomeric transporter. Effects of the E202Y mutation on transport were tested on a monomeric variant of CLC-ec1 in which a double-mutant (I201W/I422W) disrupts the homodimer interface [8]. Rigorous, complete monomer formation requires phosphatidylcholine/phosphatidylglycerol liposomes, in which transport rates are 2–4-fold slower than with *E. coli* phospholipids. (a) Representative H^+^ transport traces for WT and E202Y on the monomeric background construct. (b) Comparison of inhibitory effect of E202Y substitution on H^+^ uptake by dimeric versus monomeric transporters. (c) Cl^−^/H^+^ exchange stoichiometry (3.1, Table S2) for monomeric E202Y was determined from initial rates (dashed lines).

A strong prediction immediately arises from the E202Y structure: that inhibition by this mutation should be much less severe in a monomeric variant of the transporter, where the cross-subunit interaction between Y202 and I201 cannot exist. This prediction was tested by exploiting a monomeric CLC-ec1 construct known to support well-coupled Cl^−^/H^+^ antiport, albeit at lower absolute rates than the wild-type homodimer [8]. On this monomeric background, the E202Y substitution shows only a 4-fold reduction of H^+^ transport rate, while maintaining a Cl^−^/H^+^ exchange stoichiometry (3∶1) close to the normal value (Figure 5).

## Discussion

Ever since proton movements were understood to be coupled to anion transport in CLCs [10], attention has focused on how H^+^ navigates its way through these channels and transporters [6],[13],[22]–[24]. Macromolecular H^+^ pathways, notoriously difficult to unravel solely by experimental approaches, can often be perceived only inferentially and indirectly. In CLC-ec1, combined biochemical, electrophysiological, and structural experiments have deduced a rough trajectory for H^+^ transit through this Cl^−^/H^+^ antiporter, largely through recognition of Glu_ex_ and Glu_in_ as key dissociable residues that H^+^ transiently occupies on its way across the membrane. But details remain vague on two key issues: (1) how H^+^ gains access to the buried Glu_in_ residue from solution and (2) how it breeches the nonpolar gap between Glu_in_ and Glu_ex_.

**Figure 6:**
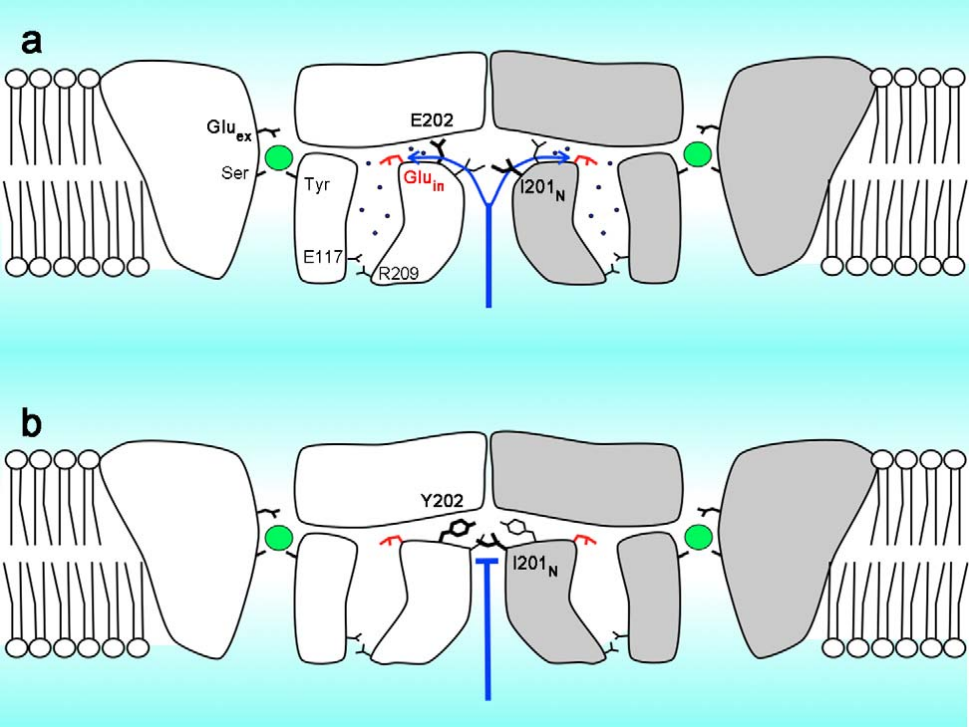
Proposed mechanism of intracellular H^+^ access. Cartoon depicts (a) homodimeric CLC-ec1, with subunits colored grey or white. In each subunit, Cl^−^ (green spheres) and crystallographic water molecules (blue dots) are shown. The proposed water-mediated interfacial H^+^ pathway connecting bulk intracellular water to the protein interior is indicated by blue arrows. Also shown are Glu_in_ (red sticks) and the serine, tyrosine, and extracellular glutamate residues that coordinate the central Cl^−^. (b) E202Y mutant, with its substituted side chain pointing out to the blocked interfacial pathway and recruiting I201_N_ (N denotes residue of neighboring subunit). Stick thickness represents vertical location of side chains. The polar pathway is also indicated as capped by the E117-R209 salt bridge.

This study establishes a specific role for E202 in the first of these proton-transfer processes. E202 stands out structurally by virtue of its strict conservation and location close to the apex of the interfacial pathway, just as it stands out functionally as the unique governor of H^+^ access to Glu_in_. The results confirm that E202 is not itself a compulsory protonation-point in the pathway and instead imply that it acts as a “water-organizer” that promotes H^+^ transfer from bulk water in the interfacial fjord to the ordered water molecules inside the protein near E202. We suppose that large nonpolar substitutions here disrupt this water conduit to Glu_in_, thereby slowing H^+^ access from intracellular solution so much that this step becomes rate-determining for overall transport. The E202Y crystal structure corroborates this idea by showing the aromatic side chain reaching across the subunit interface to interact with I201 of its homodimeric twin, thereby to plug the water-conduit. This mechanism is further validated by the minimal effect of this mutation in a monomeric variant of the transporter. Figure 6 summarizes in cartoon form the essential features of the proposal offered here for water/H^+^ access to Glu_in_.

The E202 substitutions inhibit both Cl^−^ and H^+^ in parallel, showing qualitatively that the basic coupling mechanism remains intact. However, we cannot ignore the higher Cl^−^/H^+^ stoichiometry in these slow mutants (Figure S6); this is likely not a disruption of the basic coupling mechanism but rather reflects Cl^−^ “slippage” through the inner gate in stalled transporters, which must wait for a proton to arrive before the antiport cycle can continue [14],[25]. The preservation of Cl^−^/H^+^ exchange stoichiometry in monomeric E202Y supports the idea that Cl^−^ slippage accounts for the higher Cl^−^/H^+^ stoichiometry. We emphasize that E202 is unique in controlling H^+^ access to Glu_in_; our many mutagenic maneuvers aimed at mutilating the hydrophilic character of the alternative polar pathway produced only minor effects on transport. It is worth recalling that all known CLC channels, which, like the antiporters, require transmembrane H^+^ movement for proper function [6],[22],[23] also carry the E202 equivalent; this conservation may underlie the observation that mutation of this glutamate in a mammalian CLC channel compromises its H^+^-dependent gating process [26],[27].

The internal glutamate (E203) is highly conserved among CLC antiporters, and CLC-ec1 is known to absolutely require protonation at this position for H^+^-coupled Cl^−^ movement [11],[13],[24]. But a few violations of this pattern have recently come to light among CLC antiporter homologues with nondissociable residues here [2],[28],[29]. Moreover, H^+^ transport is linked to gating of CLC channels, all of which have valine instead of glutamate at this position. These exceptions to the proton-transfer function of Glu_in_ make the strict conservation in all CLCs of the neighboring E202 position all the more notable. Thus, we imagine that while the details of H^+^ movement within the protein vary among CLC homologues, the water-organizing function of E202 proposed here for proton exchange with intracellular solvent is common to the entire superfamily.

## Materials and Methods

### Protein Purification and Crystallography

We engineered a “ΔNC” CLC-ec1 construct by deleting 15 N-terminal (residues 2–16) and four C-terminal (residues 461–464) amino acids from the natural sequence. Mutations were introduced by standard PCR-mediated cassette mutagenesis, and full coding sequences were confirmed. Expression in *E. coli* and purification of CLC-ec1 in decylmaltoside (DM) were as described [30], except that after removal of the His-tag, gel filtration (Superdex 200) replaced ion exchange chromatography as the final purification step. For crystallization, a ΔNC-Fab complex (10–20 mg/mL) [31] was mixed with an equal volume of 25%–35% PEG400, 100 mM Ca-acetate or 300 mM KCl, 100 mM tris-HCl or 100 mM Glycine-NaOH, pH 8.5–9.5. Typically, crystals were grown at 22°C in hanging or sitting drops for 2–3 wk, cryoprotected in 35% PEG400, and flash frozen in liquid N_2_. The E202Y-Fab complex was prepared as above, and crystals were formed in 38% PEG400, 200 mM CaCl_2_, 100 mM glycine-NaOH, pH 9.5. However, only the E202Y protein was found in the structure, the Fab fragment having been kicked off during crystallization.

X-ray diffraction data were collected remotely at beamline 8.2.1 or 5.0.2, Advanced Light Source. Datasets were processed and structures solved by molecular replacement as described [11]. The refined models are deposited in the Protein Data Bank (# 4ENE for ΔNC and 4FTP for E202Y).

### Cl^−^ and H^+^ Flux Experiments

Formation of liposomes reconstituted with CLC-ec1 variants (1–5 µg protein/mg lipid) and ion flux measurements have been described in detail [11]. *E. coli* phospholipids were used for all liposome experiments except with the monomeric variants, for which egg phosphotidylcholine/1-palmitoyl, 2-oleoyl phosphatidylglycerol (3/1) was used. Briefly, large multilamellar liposomes formed from several freeze-thaw cycles were extruded with a 0.4 µm filter. For H^+^ uptake, a 0.1 mL liposome sample loaded with 300 mM KCl, 40 mM citrate-NaOH, pH 4.8 was passed through a 1.5 mL Sephadex G-50 column swollen in 10 mM KCl, 290 mM K-isethionate, 2 mM glutamate-NaOH, pH 5.2, and diluted into 1.8 mL of the same solution in a stirred cell, with pH monitored continuously with a glass electrode. Cl^−^-driven H^+^ uptake was initiated by addition of 1 µg/mL valinomycin (Vln) and terminated by 1 µg/mL H^+^ ionophore FCCP. Each experiment was calibrated by addition of 50 nmoles of HCl. Cl^−^ efflux was performed similarly except that slightly different buffer systems were used. Liposomes loaded with 300 mM KCl, 25 mM citrate-NaOH, pH 4.5 were diluted as above into 1 mM KCl, 300 mM K^+^-isethionate, 25 mM citrate-NaOH, pH 4.5, Cl^−^ being monitored with an Ag/AgCl electrode. Efflux of Cl^−^ was triggered by Vln/FCCP, and at the end of the run, 30 mM β-octylglucoside was added to determine total trapped Cl^−^. Cl^−^/H^+^ stoichiometry was measured by comparison of initial slopes of H^+^ uptake and Cl^−^ efflux performed in 1–10 mM KCl, 290–300 mM K-isethionate, 2 mM citrate-NaOH, pH 5.2 using liposomes same as in H^+^ uptake experiments.

### Isothermal Titration Calorimetry

Equilibrium binding of Cl^−^ to CLC-ec1 was measured by isothermal titration calorimetry. In order to minimize Cl^−^ contamination in the protein preparations, cobalt columns charged with CLC-ec1 were washed with Cl^−^-free buffer (100 mM Na/K tartrate, 20 mM tris-SO_4_, 20 mM imidazole-H_2_SO_4_, pH 7.5, 5 mM DM) and eluted with 400 mM imidazole. Size-exclusion chromatography was in 100 mM Na/K tartrate, 10 mM tris-SO_4_, pH 7.5, 5 mM DM. Protein (150–250 µM) was titrated with 25 mM Cl^−^ solution in a Nano ITC (TA instruments) at 25°C. Data were fitted to single-site isotherms using NanoAnalyze 2.1.9 software.

## Supporting Information

Figure S1 ΔNC construct is functionally active. Cl^−^ transport (a) and active H^+^ pumping (b) of ΔNC were measured as described in Materials and Methods. Arrows mark addition of Vln, and filled circles addition of β-octylglucoside in (a) or FCCP in (b). (c) Stereo view near Cl^−^ binding site and water-filled cavity. Residues are colored in yellow (or green from other subunit), red (oxygen), and blue (nitrogen). Water molecules are displayed using small black spheres and Cl^−^_cen_ is shown as green sphere. 2F_o_-F_c_ map is contoured at 1.5 σ. (d, e) Modeled decymaltoside (DM) detergent in crystal structure of ΔNC. CLC-ec1 is drawn in surface representations with chain A in gray and chain B in sand color in side view (d) and bottom view (e). Modeled detergents are drawn as sticks, and the 2F_o_-F_c_ map around detergents is contoured at 1 σ (in blue mesh). Plugged detergent in the interfacial pathway of the A subunit is marked in red circle.
(PDF)

Figure S2 Crystallographic water molecules near Glu_in_. (a) Water molecules found in 1OTS (magenta) and ΔNC (cyan) are displayed using sphere. Cl^−^ ion is displayed in green. Stereo representations of water molecules in ΔNC (b, c, d). Atom–atom distances within 3.5 Å are displayed as red dashed lines, and distances between 3.5 Å and 4.5 Å are indicated using blue dashed lines. Water molecules were identified from maps of the B-subunit, which has systematically more crystallographic water densities.
(PDF)

Figure S3 Effect of mutations on residues lining the polar pathway. Proton transport activities from each mutant CLC-ec1 was measured from reconstituted proteoliposomes as described in the main text.
(PDF)

Figure S4 Large side chain mutations at E202 rate-limit H^+^ transport. Cl^−^ transport rates γ_o_ of the indicated E202 substitutions (filled bars) are normalized to the rate of the accompanying background construct (open bars). The completely uncoupled E148A and EAYS (E148A/Y445S) background constructs do not transport H^+^.
(PDF)

Figure S5 Stereo view of E202Y mutant near Cl^−^ binding site. Cl^−^ ion bound in Cl^−^_cen_ is shown in green. 2F_o_-F_c_ map is contoured at 1.0 σ.
(PDF)

Figure S6 Coupling of Cl^−^/H^+^ transport in E202 mutant transporters. (a) Cl^−^ (green) and H^+^ (blue) transport in indicated CLC-ec1 antiporters. Red dashed line shows initial velocity of ion movements. Scale bars indicate 100 nmole of Cl^−^ or H^+^ and 10 s. (b) Correlation plot of Cl^−^ turnover, H^+^ transport rate, and stoichiometry of Cl^−^/H^+^ transport.
(PDF)

Table S1 Data collection and refinement statistics of ΔNC and E202Y.
(PDF)

Table S2 Cl^−^/H^+^ transport metrics.
(PDF)

Table S3 Thermodynamic parameters for Cl^−^ binding.
(PDF)

Text S1 Supplementary results.
(DOCX)

## Abbreviations

DM: n-decylmaltoside
Glu_ex_: external glutamate
Glu_in_: internal glutamate
Vln: valinomycin
