# Supplementary material for: Intracellular Proton Access in a Cl−/H+ Antiporter
Source: PLoS Biol. 2012 Dec 11;10(12):e1001441. doi: 10.1371/journal.pbio.1001441 (PMC3519907; doi:10.1371/journal.pbio.1001441)
Supplement: Figure S1 — ΔNC construct is functionally active. Cl− transport (a) and active H+ pumping (b) of ΔNC were measured as described in Materials and Methods. Arrows mark addition of Vln, and filled circles addition of β-octylglucoside in (a) or FCCP in (b). (c) Stereo view near Cl− binding site and water-filled cavity. Residues are colored in yellow (or green from other subunit), red (oxygen), and blue (nitrogen). Water molecules are displayed using small black spheres and Cl− cen is shown as green sphere. 2Fo-Fc map is contoured at 1.5 σ. (d, e) Modeled decymaltoside (DM) detergent in crystal structure of ΔNC. CLC-ec1 is drawn in surface representations with chain A in gray and chain B in sand color in side view (d) and bottom view (e). Modeled detergents are drawn as sticks, and the 2Fo-Fc map around detergents is contoured at 1 σ (in blue mesh). Plugged detergent in the interfacial pathway of the A subunit is marked in red circle. (PDF) [file pbio.1001441.s001.pdf]

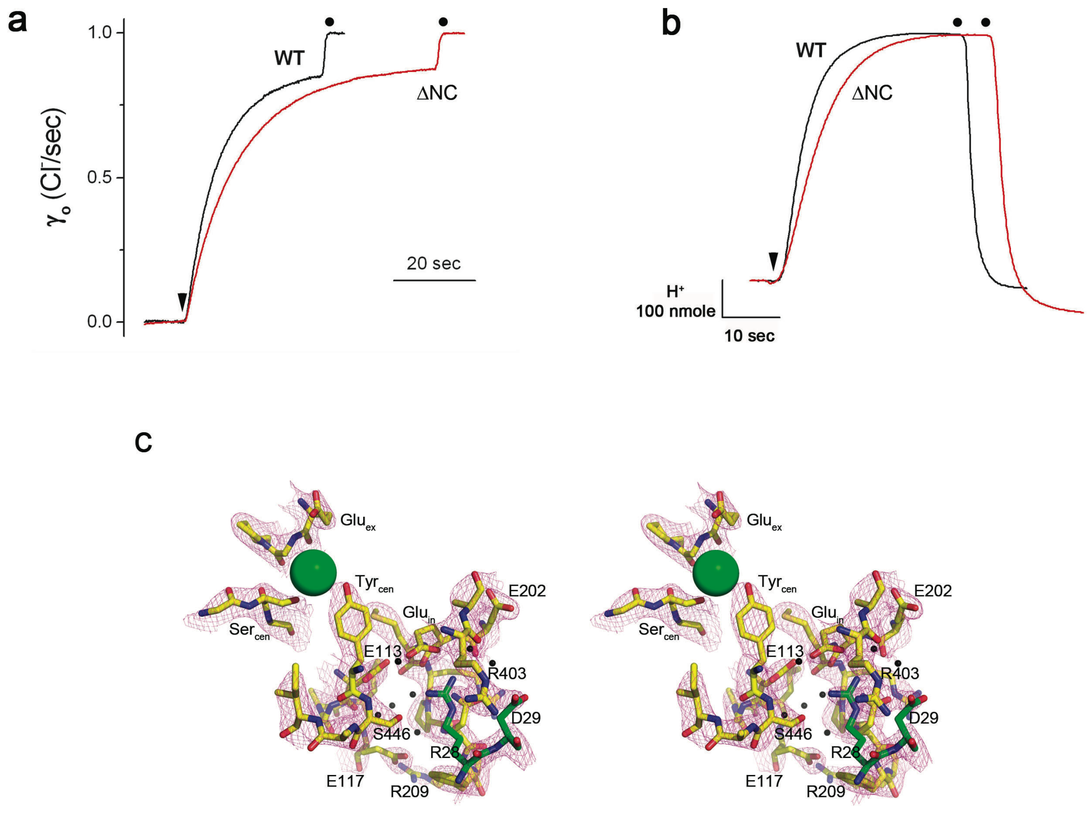

Figure S1. ΔNC construct is functionally active.

Cl^- transport (a) and active H^+ pumping (b) of ΔNC were measured as described in Methods. Arrows mark addition of Vln, and filled circles addition of β-octylglucoside in (a) or FCCP in (b). (c), Stereo view near Cl^- binding site and water-filled cavity. Residues are colored in yellow (or green from other subunit), red (oxygen) and blue (nitrogen). Water molecules are displayed using small black spheres and Cl^-_{cen} is shown as green sphere. $2F_o$-F_c map is contoured at 1.5σ.
