# Supplementary material for: Intracellular Proton Access in a Cl−/H+ Antiporter
Source: PLoS Biol. 2012 Dec 11;10(12):e1001441. doi: 10.1371/journal.pbio.1001441 (PMC3519907; doi:10.1371/journal.pbio.1001441)
Supplement: Figure S2 — Crystallographic water molecules near Gluin. (a) Water molecules found in 1OTS (magenta) and ΔNC (cyan) are displayed using sphere. Cl− ion is displayed in green. Stereo representations of water molecules in ΔNC (b, c, d). Atom–atom distances within 3.5 Å are displayed as red dashed lines, and distances between 3.5 Å and 4.5 Å are indicated using blue dashed lines. Water molecules were identified from maps of the B-subunit, which has systematically more crystallographic water densities. (PDF) [file pbio.1001441.s002.pdf]

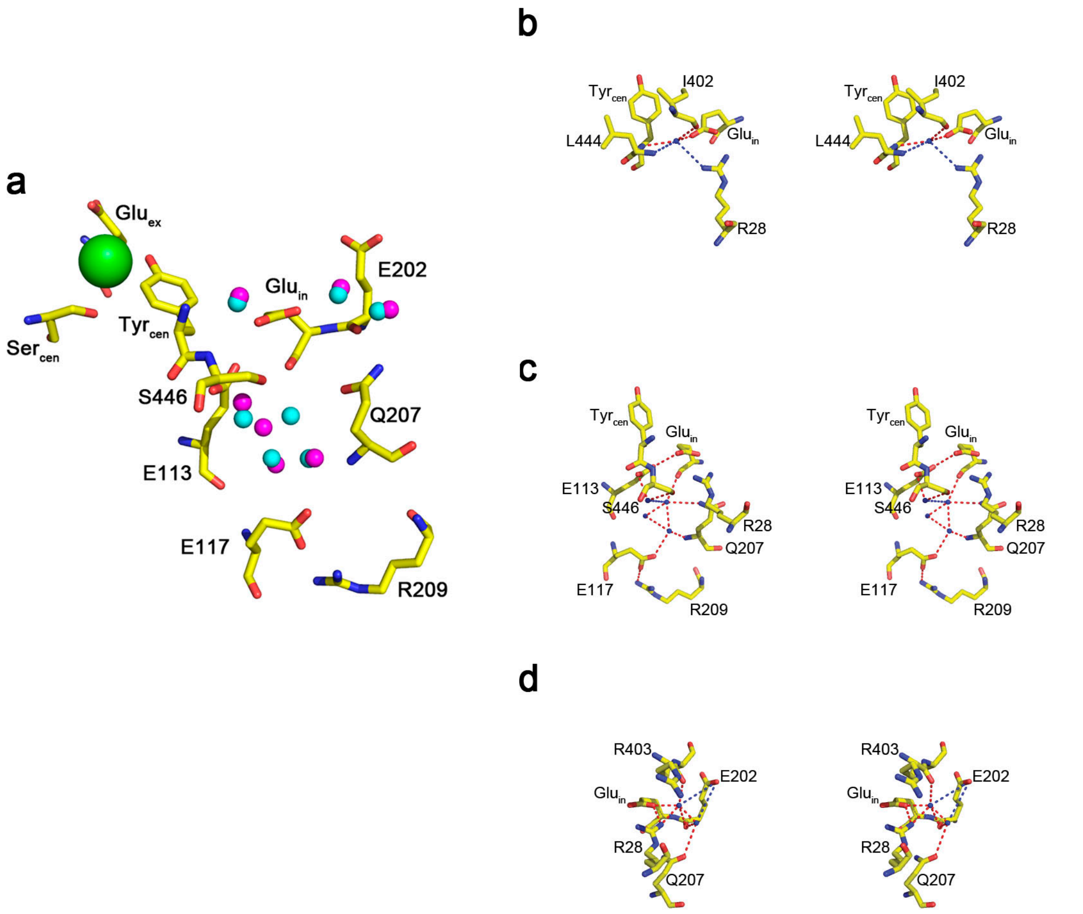

Figure S2. Crystallographic water molecules near Glu_{in}.

a. Water molecules found in 1OTS (magenta) and ΔNC (cyan) are displayed using sphere. Cl^- ion is displayed in green. Stereo representations of water molecules in ΔNC (b, c, d). Atom-atom distances within 3.5 Å are displayed as red dashed lines and distances between 3.5 Å and 4.5 Å are indicated using blue dashed lines. Water molecules were identified from maps of the B-subunit, which has systematically more crystallographic water densities.
