# Supplementary material for: Intracellular Proton Access in a Cl−/H+ Antiporter
Source: PLoS Biol. 2012 Dec 11;10(12):e1001441. doi: 10.1371/journal.pbio.1001441 (PMC3519907; doi:10.1371/journal.pbio.1001441)
Supplement: Figure S3 — Effect of mutations on residues lining the polar pathway. Proton transport activities from each mutant CLC-ec1 was measured from reconstituted proteoliposomes as described in the main text. (PDF) [file pbio.1001441.s003.pdf]

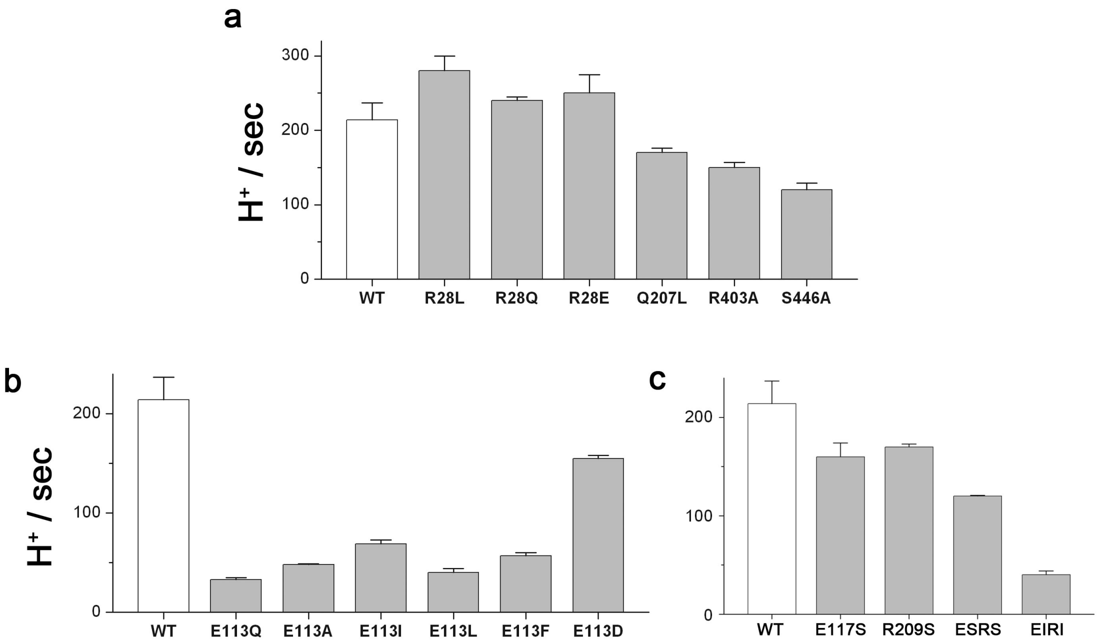

Figure S3. Effect of mutations on residues lining the polar pathway.

Proton transport activities from each mutant CLC-ec1 was measured from reconstituted proteoliposomes as described in main text.
