# Supplementary material for: Intracellular Proton Access in a Cl−/H+ Antiporter
Source: PLoS Biol. 2012 Dec 11;10(12):e1001441. doi: 10.1371/journal.pbio.1001441 (PMC3519907; doi:10.1371/journal.pbio.1001441)
Supplement: Figure S4 — Large side chain mutations at E202 rate-limit H+ transport. Cl− transport rates γo of the indicated E202 substitutions (filled bars) are normalized to the rate of the accompanying background construct (open bars). The completely uncoupled E148A and EAYS (E148A/Y445S) background constructs do not transport H+. (PDF) [file pbio.1001441.s004.pdf]

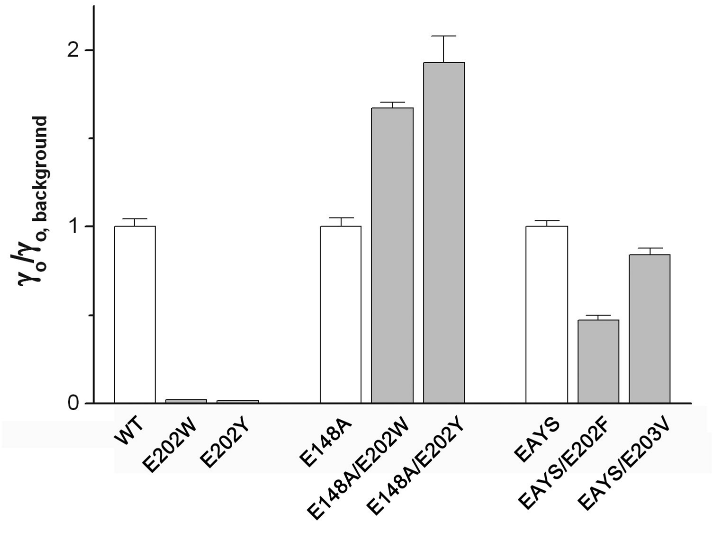

Figure S4. Large side chain mutations at E202 rate-limit H^+ transport.

Cl^- transport rates γ_o of the indicated E202 substitutions (filled bars) are normalized to rate of the accompanying background construct (open bars). The completely uncoupled E148A and EAYS (E148A/Y445S) background constructs do not transport H^+.
