# Supplementary material for: Intracellular Proton Access in a Cl−/H+ Antiporter
Source: PLoS Biol. 2012 Dec 11;10(12):e1001441. doi: 10.1371/journal.pbio.1001441 (PMC3519907; doi:10.1371/journal.pbio.1001441)
Supplement: Figure S5 — Stereo view of E202Y mutant near Cl− binding site. Cl− ion bound in Cl− cen is shown in green. 2Fo-Fc map is contoured at 1.0 σ. (PDF) [file pbio.1001441.s005.pdf]

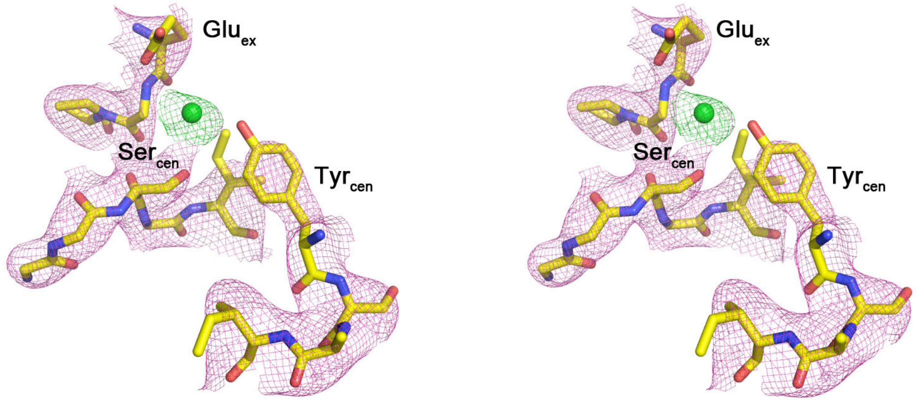

Figure S5. Stereo view of E202Y mutant near Cl^- binding site.

Cl^- ion bound in Cl^-_{cen} is shown in green. $2F_o$-F_c map is contoured at 1.0σ.
