# Supplementary material for: Intracellular Proton Access in a Cl−/H+ Antiporter
Source: PLoS Biol. 2012 Dec 11;10(12):e1001441. doi: 10.1371/journal.pbio.1001441 (PMC3519907; doi:10.1371/journal.pbio.1001441)
Supplement: Figure S6 — Coupling of Cl−/H+ transport in E202 mutant transporters. (a) Cl− (green) and H+ (blue) transport in indicated CLC-ec1 antiporters. Red dashed line shows initial velocity of ion movements. Scale bars indicate 100 nmole of Cl− or H+ and 10 s. (b) Correlation plot of Cl− turnover, H+ transport rate, and stoichiometry of Cl−/H+ transport. (PDF) [file pbio.1001441.s006.pdf]

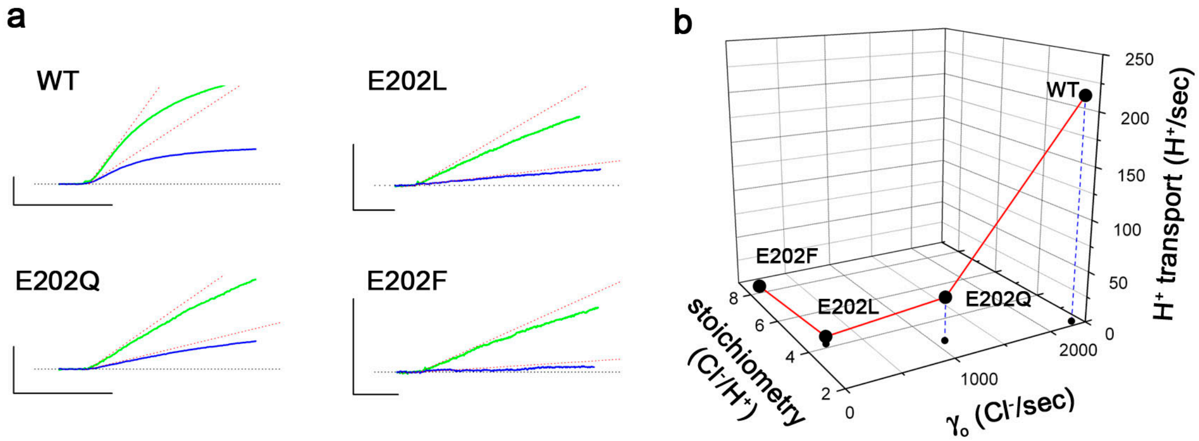

Figure S6. Coupling of Cl^-/H^+ transport in E202 mutant transporters.

a. Cl^- (*green*) and H^+ (*blue*) transport in indicated CLC-ec1 antiporters. Red dashed line shows initial velocity of ion movements. Scale bars indicate 100 nmole of Cl^- or H^+ and 10 seconds. b. Correlation plot of Cl^- turnover, H^+ transport rate, and stoichiometry of Cl^-/H^+ transport.
