# Supplementary material for: Intracellular Proton Access in a Cl−/H+ Antiporter
Source: PLoS Biol. 2012 Dec 11;10(12):e1001441. doi: 10.1371/journal.pbio.1001441 (PMC3519907; doi:10.1371/journal.pbio.1001441)
Supplement: Table S1 — Data collection and refinement statistics of ΔNC and E202Y. (PDF) [file pbio.1001441.s007.pdf]

**Table S1**. Data collection and refinement statistics of ΔNC and E202Y.

| **Data collection** | | |
|---|---|---|
| Protein | ΔNC-Fab complex | E202Y |
| Space group | C2 (a=232.3 , b=97.5 , c=173.96 ; β=132.9° ) | C2 (a=92.1 , b=84.9 , c=100.9 ; β=102.9° ) |
| Beamline | ALS 8.2.1. | ALS 5.0.2. |
| Resolution (Å)[a] | 25.0 – 2.5 (2.64 – 2.5) | 25.0 – 3.2 (3.26-3.2) |
| Unique reflections | 96398 | 12142 |
| I/σI | 10.0 (1.4) | 38.9 (5.1) |
| Multiplicity | 6.0 (6.0) | 7.3 (7.2) |
| Completeness (%) | 98.0 (97.6) | 99.0 (99.9) |
| $R_{merge}$[b] | 0.09 (1.1) | 0.16 (0.59) |
| **Refinement** | | |
| Resolution (Å) | 25.0 – 2.5 | 25.0 – 3.2 |
| Number of reflections | 96236 | 12132 |
| $R_{work}$ (%) | 23.3 | 23.4 |
| $R_{free}$ (%) | 26.5 | 31.3 |
| B factor | 49.8 | 92.1 |
| Ramachandran plot[c] | 94.0 / 5.1 / 0.7 | 89.1 / 10.0 / 0.9 |
| r.m.s.d. bond length (Å) | 0.01 | 0.01 |
| r.m.s.d. bond angle (°) | 1.3 | 1.6 |

[a] numbers in parentheses represent values for the highest resolution shell.
[b] $R_{merge} = \sum | I_i - \langle I_i \rangle | / \sum I_i$, where $\langle I_i \rangle$ is the mean intensities.
[c] percentage of residues in the favored / allowed / disallowed region.
