# Supplementary material for: Intracellular Proton Access in a Cl−/H+ Antiporter
Source: PLoS Biol. 2012 Dec 11;10(12):e1001441. doi: 10.1371/journal.pbio.1001441 (PMC3519907; doi:10.1371/journal.pbio.1001441)
Supplement: Table S2 — Cl−/H+ transport metrics. (PDF) [file pbio.1001441.s008.pdf]

Table S2. Cl^-/H^+ transport metrics

	H^+ turnover (H^+/sec)[a]	*Cl- turnover (Cl^-/sec)*[b]	*Stoichiometry (Cl^-/H^+)*[c]
Wildtype	210 ± 20 (n=7)	2300 ± 140 (n=4)	2.2 ± 0.1 (n=4)
ΔNC	200 ± 14 (n=4)	2500 ± 140 (n=4)	-
		**Polar pathway mutants**	
R28E	~250 (n=2)	-	-
R28L	~280 (n=2)	-	-
R28Q	~240 (n=2)	-	-
E113D	160 ± 3 (n=4)	-	-
E113Q	30 ± 2 (n=10)	1600 ± 30 (n=8)	4.8 ± 0.4 (n=5)
E113A	50 ± 1 (n=3)	-	-
E113I	70 ± 4 (n=3)	-	-
E113L	40 ± 4 (n=3)	-	-
E113F	60 ± 3 (n=3)	-	-
E117S	160 ± 14 (n=4)	2200 ± 110 (n=4)	-
R209S	170 ± 3 (n=4)	2000 ± 90 (n=3)	-
E117S_R209S	120 ± 1 (n=4)	1500 ± 70 (n=4)	-
E117I_R209I	40 ± 4 (n=4)	2000 ± 30 (n=4)	3.0 ± 0.1 (n=3)
Q207L	170 ± 6 (n=4)	1700 ± 40 (n=4)	-
Q207S	-	1700 ± 90 (n=3)	-
R403A	150 ± 7 (n=3)	-	-
S446A	120 ± 9 (n=3)	-	-

(*continued*)

	H^+ *turnover* (H^+*/sec*)[a]	*Cl- turnover* (Cl^-*/sec*)[b]	*Stoichiometry* (Cl^-/H^+)[c]
		**Interfacial pathway mutants**	
E202D	230 ± 12 (n=3)	-	-
E202Q	50 ± 4 (n=14)	1100 ± 60 (n=9)	3 ± 0.2 (n=4)
E202A	57 ± 5 (n=9)	800 ± 20 (n=4)	-
E202C	32 ± 5 (n=3)	-	-
E202V	16 ± 2 (n=9)	420 ± 10 (n=4)	-
E202L	8.4 ± 0.8 (n=5)	200 ± 10 (n=3)	4.1 ± 0.2 (n=4)
E202F	2.7 ± 0.2 (n=6)	130 ± 5 (n=3)	8.3 ± 0.8 (n=5)
E202H	2 ± 0.3 (n=3)	110 ± 3 (n=4)	-
E202W[d]	0.6 ± 0.1 (n=5)	50 ± 4 (n=4)	-
E202Y[d]	0.4 ± 0.1 (n=7)	40 ± 2 (n=4)	22.3 ± 1.0 (n=4)
		**H^+-transport impaired mutants** (*see Fig.3*)	
E148A	-	600 ± 30 (n=4)	-
E148A/E202F	-	300 ± 10 (n=3)	-
E148A/E202W	-	1000 ± 20 (n=3)	-
E148A/E202Y	-	1160 ± 90 (n=4)	-
EAYS (E148A/Y445S)	-	36000 ± 1300 (n=4)	-
EAYS/E202F	-	17000 ± 1000 (n=3)	-
EAYS/E203V	-	30400 ± 1300 (n=4)	-
		**Monomeric CLC-ec1**[e]	
IWIW (I201W/I422W)	110 ± 3 (n=4)	870 ± 20 (n=5)	2.3 ± 0.1 (n=4)
E202V/IWIW	28 ± 2 (n=5)	490 ± 30 (n=4)	-
E202Y/IWIW	26 ± 1 (n=4)	470 ± 10 (n=5)	3.1 ± 0.1 (n=4)

(*continued*)

Footnotes to Table S2

[a] Cl^--driven H^+ transport was measured with proteoliposomes (5 μg protein/mg lipid) loaded with 300 KCl, 40 citrate-NaOH, pH 4.8 placed in 2mL chamber containing 290 K-isethionate, 10 KCl, 2 glutamate-NaOH, pH 5.2.

[b] Passive Cl^- transport was performed in 300-fold Cl^- gradient in symmetrical pH (*inside* - 300 KCl, 25 citrate-NaOH, pH 4.5; *outside* – 300 K-isethionate, 1 KCl, 25 citrate-NaOH, pH 4.5) with proteoliposomes (1 – 5 μg protein/mg lipid)

[c] Cl^- and H^+ transport were measured in identical experimental conditions (*inside* – 300 KCl, 40 citrate-NaOH, pH 4.8; *outside* – 300 K-isethionate, 1KCl, 2 citrate-NaOH, pH 5.2), and stoichiometry is reported as ratio of initial rates.

[a,b] All the values were obtained by initial velocity measurements [14].

[d] H^+ turnover rates for these mutants were calculated by linear fit of data at early times.

[e] Proteoliposomes were prepared at 1μg protein/mg lipid density in 3:1 (*w/w*) mixture of egg PC and POPG lipids.
