# Supplementary material for: Intracellular Proton Access in a Cl−/H+ Antiporter
Source: PLoS Biol. 2012 Dec 11;10(12):e1001441. doi: 10.1371/journal.pbio.1001441 (PMC3519907; doi:10.1371/journal.pbio.1001441)
Supplement: Table S3 — Thermodynamic parameters for Cl− binding. (PDF) [file pbio.1001441.s009.pdf]

Table S3. Thermodynamic parameters for Cl^- binding.

	Wildtype (n=3)	E202Y (n=3)
Number of site	1	1
K_d (mM)	0.74 ± 0.013	1.6 ± 0.15
ΔG^o (kcal/mol)	-4.3 ± 0.01	-3.8 ± 0.06
ΔH^o (kcal/mol)	-5.0 ± 0.04	-1.8 ± 0.12
