# Supplementary material for: Intracellular Proton Access in a Cl−/H+ Antiporter
Source: PLoS Biol. 2012 Dec 11;10(12):e1001441. doi: 10.1371/journal.pbio.1001441 (PMC3519907; doi:10.1371/journal.pbio.1001441)
Supplement: Text S1 — Supplementary results. (DOCX) [file pbio.1001441.s010.docx]

**Supplementary Materials Text S1**

**Supplementary Results**

*Crystallographic water molecules near Glu_in_.*

A crystal structure of wildtype CLC-ec1 at 2.5Å (PDB #1OTS) shows several water molecules near Glu_in_^1^. In order to verify structural water in this region, we crystallized and solved the structure of an engineered protein, ΔNC (PDB #4ENE) at 2.4 Å, which is functionally indistinguishable from wildtype (Fig. S1a,b). We carefully built water molecules in prominent positive densities from F_o_-F_c_ maps, considering local chemistry such as polarities and atom clashes (Fig. S2). Chain B has 34 modeled water molecules, while chain A has only 18. One of these makes a short (2.5 Å) H-bond to the carboxylate of Glu_in_, forms a H-bond to the amide backbone of Y445, and could be stabilized by the backbones of I402 and L444 and the side chain of R28 (Fig. S2b). Four additional water molecules, within H-bonding distance of each other, reside in the small water-filled cavity lined by Glu_in_, R28, E113, Q207, S446, E117 and R209 (Fig. S2c). Two water molecules located between E202 and Glu_in_ form complex H-bonding networks with side chains of neighboring residues such as E202, Glu_in_, R28, and Q207 and carbonyl backbone of E202 and R403 (Fig. S2d). These waters could in principle participate in separate H^+^-transport pathways from intracellular aqueous solvent to Glu_in_; “polar” and “interfacial” pathways (Fig. 1b and Fig. S1c).

We modeled detergent molecules around the solvent-lipid interface of CLC-ec1, as 6 DM molecules with maltose headgroups and alkyl chains, and 3 with only the maltose headgroup. Interestingly, one of the maltose moieties is plugged into the interfacial pathway of chain A (Fig. S7), but that pathway is free of detergent in chain B. This can account for the smaller number of crystallographic water molecules detected in chain A.

*Effects of mutations of residues lining the polar pathway.*

The polar pathway region of CLC-ec1, which separates Glu_in_ from the cytoplasmic solution, contains a cluster of polar residues, R28, E113, E117, E202, Q207, R209, R403, and S446 (Fig. 1b and Fig. S1c). We examined in particular a salt-bridge between R28 and E203, which previous mutagenesis studies^2,3^ showed not to be necessary for properly coupled Cl^-^/H^+^ transport; that work, however, did not measure H^+^ transport rates in R28 mutants. As shown in Fig. S3a, H^+^-transport rates are unaffected by substitution of R28 with glutamate, glutamine, leucine, a loud negative result that dispels any notion that the R28-Glu_in_ salt bridge participates in the transport mechanism. Disruption of another salt-bridge (D29 and R403) closely located near Glu_in_ by the R403A mutation also preserve robust H^+^ transport. Mutations of other polar pathway residues, Q207L and S446A, show < 2-fold decreases of H^+^-transport rates. The E113Q mutation, on a glutamate near to Glu_in_, produces a 6-fold decrease (Fig. S3b), while the E113D substitution leaves H^+^-transport unaffected. A variety of substitutions at E113 preserve H^+^-coupled antiport (Fig. S3c) at 15-30% of wildtype rates, a result that rules out this residue as mechanistically essential.

E117 and R209 form a salt bridge that physically isolates the water-filled cavity in the polar pathway from bulk water and so might act as an entry point for H^+^ access to Glu_in_. But a variety of electrostatically disruptive mutations here, including the double-Ile substitution, fail to slow H^+^ transport by more than 5-fold (Fig. S3d).

*Mechanistic target of E202 mutations*

As described in the main text, substitution of bulky side chains at E202 makes H^+^ transport rate-limiting in the antiport cycle, as shown by only small effects of such mutations on Cl^-^ transport in the E148A background, which does not transport protons. This result is confirmed by a similar test performed on a different uncoupled mutant, E148A/Y445S, on which background the E202F mutation slows Cl^-^ transport only 2-fold (Fig. S4).
